# Active learning with non-*ab initio* input features toward efficient CO_2_ reduction catalysts†
†Electronic supplementary information (ESI) available. See DOI: 10.1039/c7sc03422a


**DOI:** 10.1039/c7sc03422a

**Published:** 2018-04-17

**Authors:** Juhwan Noh, Seoin Back, Jaehoon Kim, Yousung Jung

**Affiliations:** a Graduate School of EEWS , Korea Advanced Institute of Science and Technology (KAIST) , 291 Daehakro , Daejeon 305-701 , Korea . Email: ysjn@kaist.ac.kr ; Tel: +82-042-350-1712

## Abstract

In a conventional chemisorption model, the d-band center theory (augmented sometimes with the upper edge of the d-band for improved accuracy) plays a central role in predicting adsorption energies and catalytic activity as a function of the d-band center of solid surfaces, but it requires density functional calculations that can be quite costly for the purposes of large scale screening of materials. In this work, we propose to use the d-band width of the muffin-tin orbital theory (to account for the local coordination environment) plus electronegativity (to account for adsorbate renormalization) as a simple set of alternative descriptors for chemisorption which do not require *ab initio* calculations for large-scale first-hand screening. This pair of descriptors is then combined with machine learning methods, namely, neural network (NN) and kernel ridge regression (KRR). We show, for a toy set of 263 alloy systems, that the CO adsorption energy on the (100) facet can be predicted with a mean absolute deviation error of 0.05 eV. We achieved this high accuracy by utilizing an active learning algorithm, without which the accuracy was 0.18 eV. In addition, the results of testing the method with other facets such as (111) terrace and (211) step sites suggest that the present model is also capable of handling different coordination environments effectively. As an example of the practical application of this machine, we identified Cu_3_Y@Cu* as an active and cost-effective electrochemical CO_2_ reduction catalyst to produce CO with an overpotential ∼1 V lower than a Au catalyst.

## Abstract 2

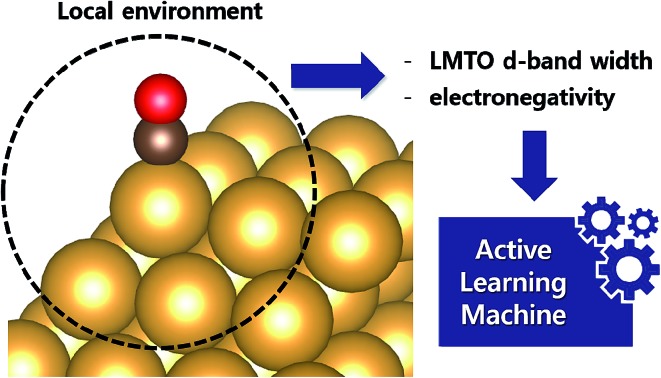
In this work, we propose the use of the d-band width of the muffin-tin orbital theory (to account for the local coordination environment) plus electronegativity (to account for adsorbate renormalization) as a simple set of alternative descriptors for chemisorption which do not require *ab initio* calculations for large-scale first-hand screening.

## 1. Introduction

Understanding and predicting the energetics associated with bond-forming and bond-breaking reactions occurring on the surface of solid materials is the central theme of heterogeneous catalysis research. Among many other catalysis theories, in particular, the Sabatier principle^1^ is an important simple concept that states that the chemisorption strength of key reaction intermediates on catalyst surfaces should be just right to maximize catalytic activity; either too weak or too strong binding leads to insufficient activation of the reactant or great difficulty in product desorption after catalysis completion, respectively, and therefore a typical volcano activity relationship can be plotted as a function of the binding energies.^2^ In a series of pioneering studies, Nørskov and co-workers suggested a way to understand the chemisorption of reaction adsorbates in terms of the surface electronic structure of the materials in a so-called d-band theory.^3^ Here, the essence is that the binding energy of an adsorbate to a metal surface is largely dependent on the electronic structure of the surface itself, namely, the d-band centre of the surface rather than the entire detailed density of states (DOS).

With the recent progress in electronic structure methods (mainly density functional theory calculations for solids) that can now give reliable electronic structures and binding energetics, the d-band center theory, along with the scaling relations that exist between the binding energies of related adsorbates, has been successfully applied to understanding and predicting new materials for many different applications.^4^–^8^ However, exceptions were also found in which the usual d-band center trend could not explain the activity measured.^9^–^11^ The main cause for the aforementioned exceptions was the lack of consideration of the spread in energy states and, for those cases, the correlation between the d-band center and the activity was improved by the introduction of the d-band width (*W*_d_)^11^ and the upper edge of the d-band (*E*_u_) when using the Hilbert transform of the projected DOS.^10^ It has also been suggested that the standard d-band model is not a reliable measure for systems such as the Pt–Au–M ternary nanoparticle system because of the notable change in the electronic structure caused by the strain and ligand effects compared to that for pure Pt nanoparticles.^12^

Recently, instead of energetic descriptors, an alternative metric to describe the activity of the catalyst based on the local geometric features of the active sites has been proposed, namely, the generalized coordination number.^13^,^14^ This approach yielded simple coordination–activity plots that predicted the optimal geometric structure of platinum nanoparticles, which were then experimentally verified.^15^ Nonetheless, these generalized coordination numbers are not easy to extend to alloy systems, at least in their current form, since they cannot distinguish the different electronic structures associated with the different metal atoms in the alloys. In this sense, it would be helpful to have a descriptor that can describe the local coordination environment as well as the electronic structure of the constituent metal atoms when describing metal alloys. Indeed, an approach to satisfying the latter two aspects has been proposed in an orbital-wise coordination number^16^ although it still requires *ab initio* calculated geometries to obtain high accuracy.

We note that an open database such as the Materials Project^17^ provides an excellent general platform to perform large scale material screening for many applications using various DFT-derived quantities such as the density of states (DOS). In this work, we propose to use the d-band width within the (tight binding) linear muffin-tin orbital (LMTO or TB-LMTO) theory^18^ as a simple non-*ab initio* quantity that can be efficiently used in first-stage catalyst screening applications. Unlike the usual d-band width obtained from DFT calculations that is a bulk property of the slab, the LMTO d-band width in practice can capture the local electronic structure due to the truncation of the interatomic couplings up to the second nearest neighbor atoms.

Using this LMTO-based d-band width, we construct, as a toy problem, a chemisorption model to compute the binding energy of *CO on various metal alloys. The idea is to establish a functional relation between the simple yet analytical LMTO d-band width and *CO binding energy, and to perform large-scale screening using these non-*ab initio* descriptors to find a material with optimal *CO binding for an efficient CO_2_ reduction reaction (CRR). Although there are many linear models between the descriptors such as the d-band center (sometimes augmented by the upper edge of the d-band) and the binding energy of the adsorbate,^10^,^11^,^19^ to increase the prediction accuracy we adopt machine learning techniques to incorporate the potential nonlinear correlation between the descriptors and binding energies.

We note that there are two machine learning models to predict *CO binding energy in literature that inspired the present work and used simple descriptors, with 13 electronic structure based descriptors in one case^20^ and 2 local geometric features in the other case.^21^ The authors, in both reports, obtained a similar mean absolute deviation error of 0.13 eV when predicting the *CO binding energy for various alloy systems despite the very different input features (13 electronic *vs.* 2 geometric), demonstrating the importance of proper feature selection for improved learning efficiency. It can also be noted that the input features in both machines still required *ab initio* calculations to relax the geometries and/or to obtain accurate electronic structures of the materials. In a recent and very interesting reaction network study,^22^ non-*ab initio* extended connectivity fingerprints (ECFPs) based on a Gaussian process (GP) model were used to predict the formation energies of ∼90 surface intermediate species, with a final goal of identifying the most likely reaction pathways of syngas formation on rhodium (111), although a potential transferability limitation of ECFPs for different adsorption sites was noted in which *ab initio* calculations would still be needed. Finding non-*ab initio* input features that represent local environments is thus of significant current interest in the field of heterogeneous catalysis.

In this work, we propose the use of two non-*ab initio* input features, *i.e.* LMTO d-band width and electronegativity, as an easy-to-compute model to predict the *CO binding energy of various alloy systems. By combining the aforementioned descriptors and utilizing the latest active learning algorithms, we obtained a root mean square error (RMSE) of 0.05 eV. As an example of the application of the machine to screen transition metal based alloy catalysts for CRR, we identified three promising catalysts, Cu–Fe@Cu, Cu_3_Sc@Cu* and Cu_3_Y@Cu*, with higher activity than the most active Au based catalysts.

## 2. Methods

### 2.1 Non-*ab initio* descriptors for chemisorption

Selecting proper descriptors is one of the most important tasks in machine learning since it determines the learning efficiency as well as the prediction power. While most current descriptors for chemisorption models in machine learning require *ab initio* calculations, such as the d-band center and its higher-order moments,^21^ our main focus is to utilize non-*ab initio* based descriptors. As proposed by Nørskov and co-workers,^3^ the surface–adsorbate binding process can be decomposed into two effects; the coupling of the adsorbates with the sp-bands and d-bands of the catalyst surface. For the former, based on an empirical correlation between the sp-coupling and surface–adsorbate bonding distance,^19^,^23^–^25^ the sp-coupling term is usually estimated as the geometric mean of the electronegativity for the first coordination shell 
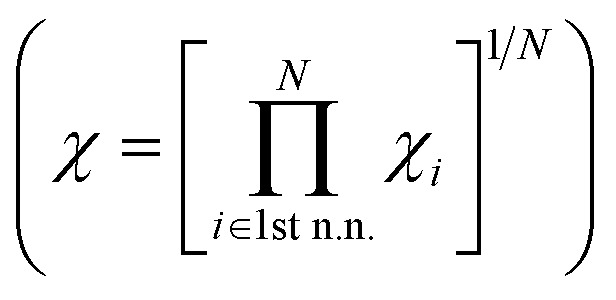
 and we followed the same practice. For the latter, instead of the conventional *ab initio* d-band center, we propose the use of a non-*ab initio* analytical expression of the d-band width within the LMTO theory, denoted *W*LMTOd (see eqn (S4) in the ESI†). While *W*LMTOd does not contain information about the center position of the d-band, there are two advantages of using it as a descriptor for the purpose of large-scale screening. Firstly, it effectively captures the local chemical environment of the d-state for chemical events due to the use of interatomic coupling terms with the second nearest neighbor atoms from the active site, a similar concept as used for the generalized coordination number.^13^,^14^,^26^ Secondly, it does not require DFT calculations to generate input features since the analytical expression for *W*LMTOd can be evaluated based on tabulated values for a given composition (see ESI†). Since the learning will be supervised by labeled reference data, one would still need the DFT calculations of the *CO binding energy to establish a training set. However, we emphasize that, unlike existing *ab initio* input features such as the d-band center, the current model does not require additional DFT calculation to predict the *CO binding energy once the machine is trained.

### 2.2 Supervised (active) learning methods

Supervised machine learning is a method used to predict a target value (*e.g.* total energy) from given inputs (*e.g.* electron density). Among many algorithms, we used two machine learning methods, artificial neural network (ANN or simply neural network, NN) and kernel-ridge-regression (KRR) methods. NN involves a stack of layers consisting of input, (multiple) hidden and output layers, and each layer contains many neurons. The network is trained by measuring and minimizing the errors between the predicted output and reference values (called labels) using backpropagation algorithms.^27^ In KRR, the model is trained by solving the ridge regression with kernel-function-based non-linear transformation to input data. A kernel function is used to transform the original input data into an easy-to-train form by describing the relation (or distances) between the input data.^28^ Excellent reviews introducing machine learning algorithms for additional details can be found elsewhere.^28^,^29^

As briefly introduced, in supervised machine learning, most of the computational cost of building the model usually occurs when generating the reference data in the training set and running the cost-function minimizations. Therefore, it is of significant practical interest to reduce the training set size to as small as possible without compromising the representability of the system. Active learning is an algorithm in which the machine can point out samples with maximal information about the target function,^30^ and it is widely used currently in classification/filtering, speech recognition, information extraction, computational biology, *etc.*, for example.^31^ In this work, we utilize active learning in the design of the catalyst to choose the minimal list of samples for training that can represent the given class of alloy material.

We used two types of machine learning methods, neural network (NN) and kernel-ridge-regression (KRR) methods, as described in detail in the Computational details section. For active neural network learning,^30^ we used an ensemble NN model. In this method, one constructs multiple NN models in an ensemble (5 in our case) based on the same training set but optimized with different initializations, identifies examples in the test set characterized by the largest variance (or ambiguity) within the ensemble and then includes these new examples in the next training set for further learning. Since this algorithm does not require a label (*CO binding energy), we denote this method an ensemble NN without labels. If one already has labels for the test set, improved accuracy might be expected by computing the actual residual, or error, (the difference between the ensemble-averaged model-predicted values and the true labels) and identifying an example with the largest residual. We denote this an ensemble NN with labels.

For KRR, since there are analytical unique solutions for given training samples and hyper-parameters, methods similar to ensemble NN cannot be constructed. Instead, there are other types of active learning algorithm for KRR in literature,^32^–^35^ and in this work, we used the residual regression model. In this algorithm, one first obtains a *CO binding energy predictor with the training set (as in conventional KRR) with a certain training set error. Next, one constructs an error predictor with the same training set using the previous training set error as output data. This error predictor is then used to identify the samples that are the most different from the existing training set. In other words, one estimates the errors of all the test samples using this error predictor and can find samples with the lowest absolute value of the generated outputs for further learning. Since this algorithm does not need the labels of the test set, we denote this method active KRR without labels.^34^ For a similar reason to that considered for ensemble NN with labels, since in the present case all of the labels of the test set are available, we also constructed another active KRR model utilizing the labels of the test set, denoted active KRR with labels. Here, one includes samples with the highest absolute value of the residual of the error predictor on the test set in the next training set. The residual of the error predictor is defined as the difference between the output of the error predictor and the error calculated from the *CO binding energy predictor.

## 3. Models and computational detail

### 3.1 Descriptor evaluations

**Fig. 1:**
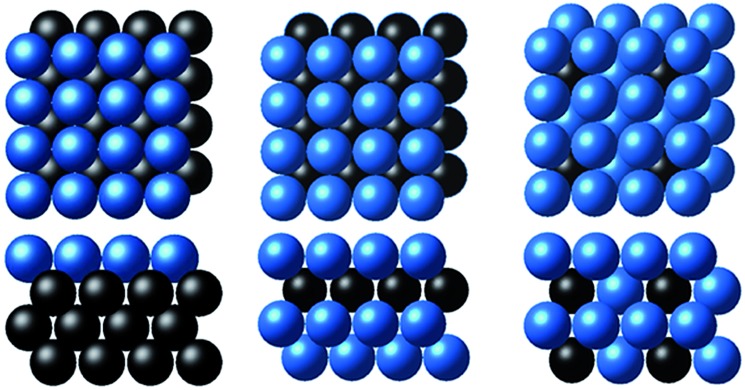
The three subsurface alloy models considered in this study (from left to right): X@M, M–X@M and M_3_X@M (the blue and black balls denote M and X metals, respectively).^20^,^21^

As a toy problem of predicting the *CO binding energy on the fcc(100) slabs, we considered surface overlayers in the form of X@M, M–X@M and M_3_X@M, where M = Ag, Au, Cu, Ni, Pd or Pt and X is the 3d, 4d or 5d transition metal (263 samples in total), as shown in Fig. 1 and taken from ref. 20 and 21. The calculated descriptors (*χ* and *W*LMTOd) for machine learning for the latter set are listed in Table S3 of the ESI.†

Counting of the first and second nearest neighboring atoms was defined layer by layer using the two topmost metal layers. In other words, for the first layer, around the binding site, there are 4 atoms of the nearest and 4 atoms of the second nearest neighbors on the basis of distance from the binding site. Similarly, this can be applied to the second layer; the number of the first and second nearest neighboring atoms around the binding site in the second layer is 4 and 8. Using this definition as the coordination number, *χ* was calculated on both the Mulliken (*χ*_M_) and Pauling (*χ*_P_) scales. The estimation of *W*LMTOd is described in detail in the ESI,† but we emphasize that *W*LMTOd can be obtained without *ab initio* geometry relaxations, unlike in previous approaches^20^, since the interatomic distances of the alloy models are estimated using Vegard’s law^19^ for the two topmost layers. More about these calculations is shown in detail in the ESI.†

The other quantities used for comparison and training/testing such as *d*_c_, *W*cald and *CO adsorption energy (*E*_CO,cal_) are taken from ref. 20. The upper edge of the d-band (*E*_u_) is defined as *d*_c_ + *W*_d_/2, and for clarity, *E*LMTOu is defined as *d*_c_ + *W*LMTOd/2 and *E*calu is defined as *d*_c_ + *W*cald/2.

### 3.2 Machine learning models

For all of the NN models, a MATLAB R2015b Neural Network Toolbox™^36^ was used with a Parallel Computing Toolbox™.^37^ For the training algorithm, a Levenberg–Marquardt training algorithm,^38^–^40^ a kind of backpropagation algorithm, was used with a hyperbolic-tangent activation function. For conventional ANN, both single hidden layer (SHL ANN) and double hidden layer (DHL ANN) models were tested. The SHL ANN was trained with the number of nodes in the hidden layer increasing from 4 to 20, and for the DHL ANN a second hidden layer with 4 nodes was added to the SHL ANN. The total of 263 data samples were randomly divided into three parts; training, validation and test sets with a ratio of 60 : 15 : 25.

To implement the ensemble NN, 5 independent DHL ANN models were constructed with 4 samples randomly chosen as an initial training set. During the active learning process, 2 additional samples were identified in each iteration from the untrained samples and added to the training set until the final training set reached 60% of the total data.

For all of the KRR models, the conventional KRR method (non-active KRR)^29^ with a kernel function, *k*(*x*_*u*_,*x*_*v*_) = exp(–‖*x*_*u*_ – *x*_*v*_‖_2_/*σ*), was used.

To reduce the computational cost of the hyper-parameter optimizations, we explicitly fixed the kernel width as *σ* = 0.5 and the regularization factor to be 0.005. For the active KRR models, the same procedure as that of ensemble NN was applied. For all machine learning models, 4800 independent trials were applied to remove randomness from the initial training sets.

### 3.3 DFT calculations

We performed DFT calculations for selected candidates using the Vienna Ab initio Simulation Package (VASP)^41^ with a projector-augmented wave (PAW)^42^ and the revised Perdew–Burke–Ernzerhof (RPBE) exchange–correlation functional.^43^,^44^ The energy cut-off for the plane-wave basis set was 500 eV, and *k*-points were sampled using a (8 × 4 × 1) Monkhorst–Pack mesh.^45^ We modelled the fcc(100) slabs with a (4 × 2) atom containing surface unit cell and 4 layers. A 15 Å vacuum was used to minimize the interaction between periodic images in the *z*-direction. The topmost 2 layers were relaxed until the residual force on each atom became less than 0.05 eV Å^–1^. The free energy of the reaction intermediates on the surface was obtained by using a harmonic oscillator approximation at 298.15 K implemented using an Atomic Simulation Environment (ASE) program,^46^ and the free energy of gas molecules was obtained using an ideal gas approximation at 298.15 K implemented using ASE. To correct the systematic DFT errors for describing C=O double bonds and H–H bonds, we added a +0.15 eV correction for each C=O bond, *i.e.* +0.30 eV for a CO_2_ molecule and +0.15 eV for an adsorbed *COOH, and +0.10 eV for the H–H bond in a H_2_ molecule.^47^ We also applied approximate solvation corrections for *CO (–0.10 eV) and *COOH (–0.25 eV) to account for the effect of solvation.^48^

## 4. Results and discussion

**Fig. 2:**
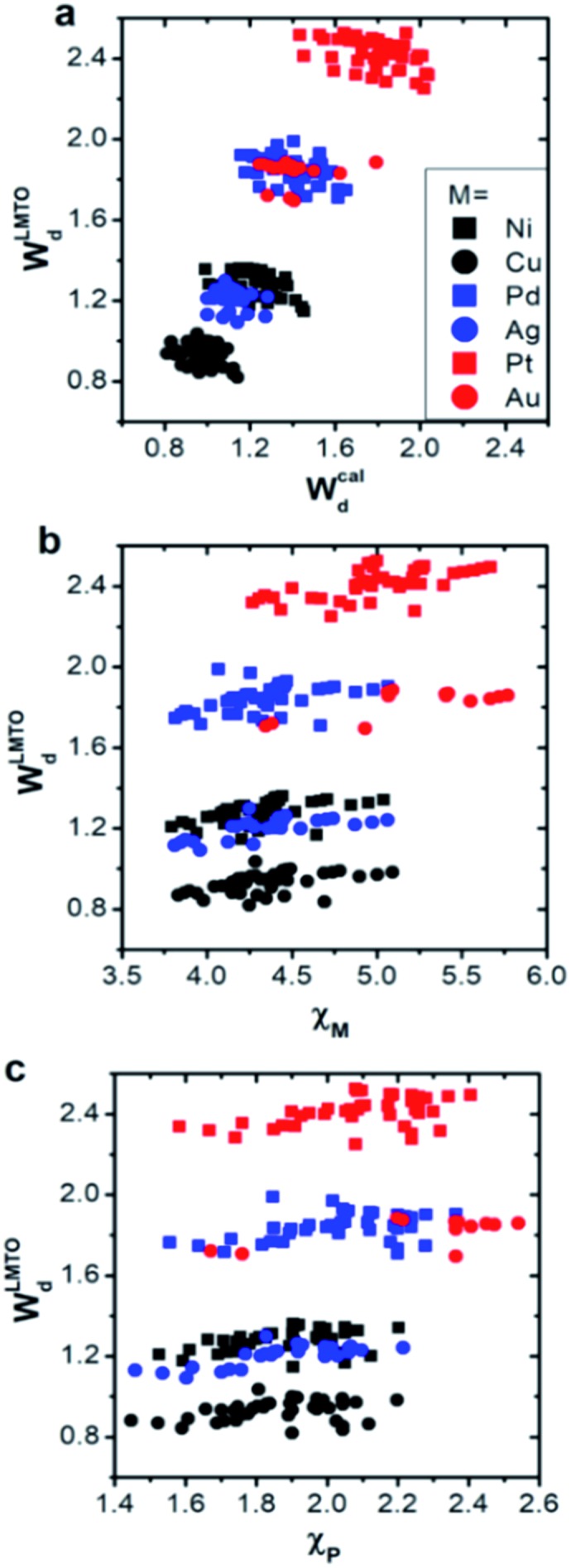
(a) Comparison of the LMTO-based d-band widths (*W*LMTOd) to the DFT calculated values (*W*cald) for various alloy models: X@M, M–X@M and M_3_X@M (see Computational details section). The d-band widths are normalized to 1 for pure Cu for easy comparison. The data distribution of *W*LMTOd*versus* two types of electronegativity, (b) Mulliken *χ*_M_ and (c) Pauling *χ*_P_.

We first compared the LMTO-based d-band widths, *W*LMTOd, to the DFT d-band widths, *W*cald. Two main points can be drawn from the results shown in Fig. 2a. Firstly, *W*LMTOd shows the same qualitative trend as *W*cald. Secondly, many different materials are clustered around similar *W*LMTOd or *W*cald values, but they are largely resolved by introducing both *χ*_M_ and *χ*_P_ as shown in Fig. 2b and c.

Similar to the correlation between the DFT and LMTO-based d-band widths shown in Fig. 2a, there are also several studies showing that the electronic property from LMTO theory is strongly correlated to that from DFT calculation. As reported by A. Vojvodic *et al.*,^11^ the d-band center approximated by LMTO theory showed the same trend as that from DFT calculation. In addition, as reported by J. R. Kitchin *et al.*,^49^ it was theoretically shown that the matrix element, defined as the summation of the interatomic coupling terms up to all the nearest neighbors, from LMTO theory is strongly correlated to the d-band width from DFT calculations. All of these results suggest that the proposed d-band width using LMTO theory can reasonably represent the electronic properties of local environments.

**Fig. 3:**
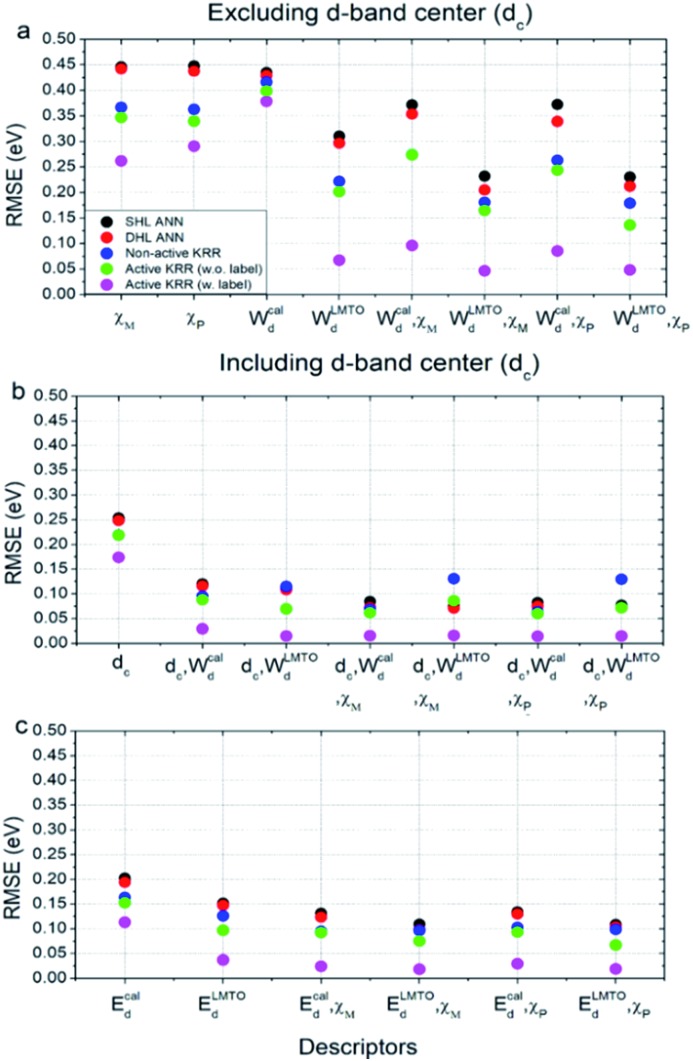
The performance of various machine learning models with different descriptors: (a) without an *ab initio* d-band center, and (b and c) with a d-band center. All RMSE values were calculated for the entire data set.

**Fig. 4:**
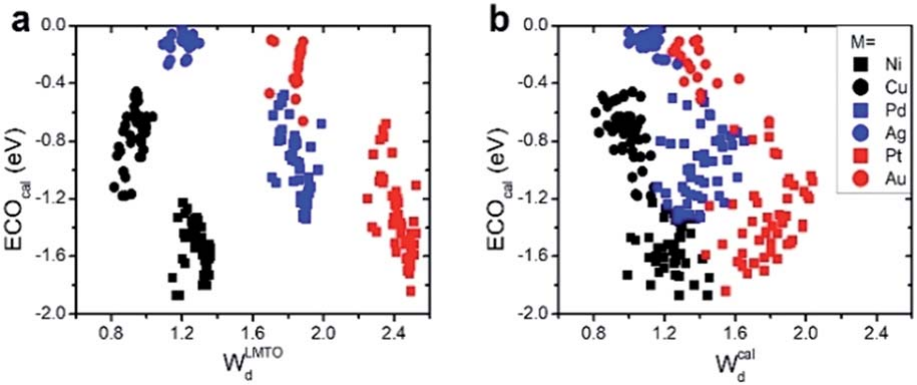
Data distributions of *CO binding energies *versus* (a) *W*LMTOd and (b) *W*cald.

Using these two selected descriptors, the performances of various machine learning models are summarized in Fig. 3. Three points are noteworthy:

(1) Interestingly, even the LMTO-based d-band width (*W*LMTOd) alone performs quite well (RMSE = 0.07 eV). In addition, the LMTO-based d-band width consistently yields a lower RMSE than the *ab initio*-based d-band width by 0.05–0.15 eV, when combined with *χ*. This suggests that the local concept involved in the evaluation of *W*LMTOd (interactions up to the 2^nd^ nearest neighboring atoms in the surface and subsurface layers) helps to correlate better with the binding affinity as compared to the bulk surface quantity (*W*cald). The localized nature of *W*LMTOd can also be confirmed by the data shown in Fig. 4, in which the core@M alloys with the same M species are all clustered around a similar region, whereas the distribution of *W*cald is much broader for the same M species. This clustering is a helpful feature for active learning since it becomes easier to choose new data which differ the most from the existing training set data.

(2) For all of the combinations of descriptors shown in Fig. 3a, KRR (0.05–0.37 eV) performs consistently better than ANN (0.21–0.45 eV) for the chemistry studied.

(3) The actively learned KRR enhances the accuracy of the model significantly, lowering the RMSE by 0.13 eV compared to that of conventional KRR (from 0.18 to 0.05 eV) for the best case. A more detailed discussion on the effects of active learning on the RMSE variance and accuracy for both ANN and KRR will be given later.

Therefore, by combining all of these results, the best chemisorption model without any *ab initio* inputs is the active KRR model based on the pair of descriptors (*W*LMTOd and *χ*_P_) with an RMSE of 0.05 eV. This can be compared with previous results (0.13 eV) using ANN with *ab initio* based parameters and geometries.

There are many indications that the d-band center alone is not a sufficient descriptor for more complicated catalyst structures,^9^–^12^ but the d-band center is still one of the most widely-used descriptors for chemisorption models on the catalyst surface, so we also considered models that included the conventional d-band center (Fig. 3b and c). As expected, for all of the machine learning methods and combinations of descriptors, the inclusion of the d-band center improves the accuracy significantly, with the best model being the active KRR with any combination of descriptors with RMSE ∼ 0.02 eV. It is remarkable, however, to note that the difference between the cost-effective LMTO d-band width model (0.05 eV) and the expensive d-band center model is only 0.03 eV.

Because the current model is trained using a single type of active site (fcc(100)-terrace), we tested the extensibility of the approach by treating two different additional coordination environments, namely fcc(111)-terrace and fcc(211)-step sites. For each fcc surface type, 3 kinds of surface slab model (X@M, M–X@M and M_3_X@M where M, X = Ni, Cu, Pd, Ag, Pt or Au) were considered. We constructed the machine based on the active KRR with labels model with a pair of descriptors (*W*LMTOd and *χ*_P_) and a total of 305 samples. We obtained an overall combined RMS error of 0.08 eV, quite close to the 0.05 eV obtained with the (100) facet only, as shown in Fig. 3a. Apparently, the latter subset is not an extensive test of the method, yet it clearly shows the reasonable potential of the d-band width as an efficient descriptor for the first-hand screening of large candidates before DFT calculation on various coordination environments.

**Fig. 5:**
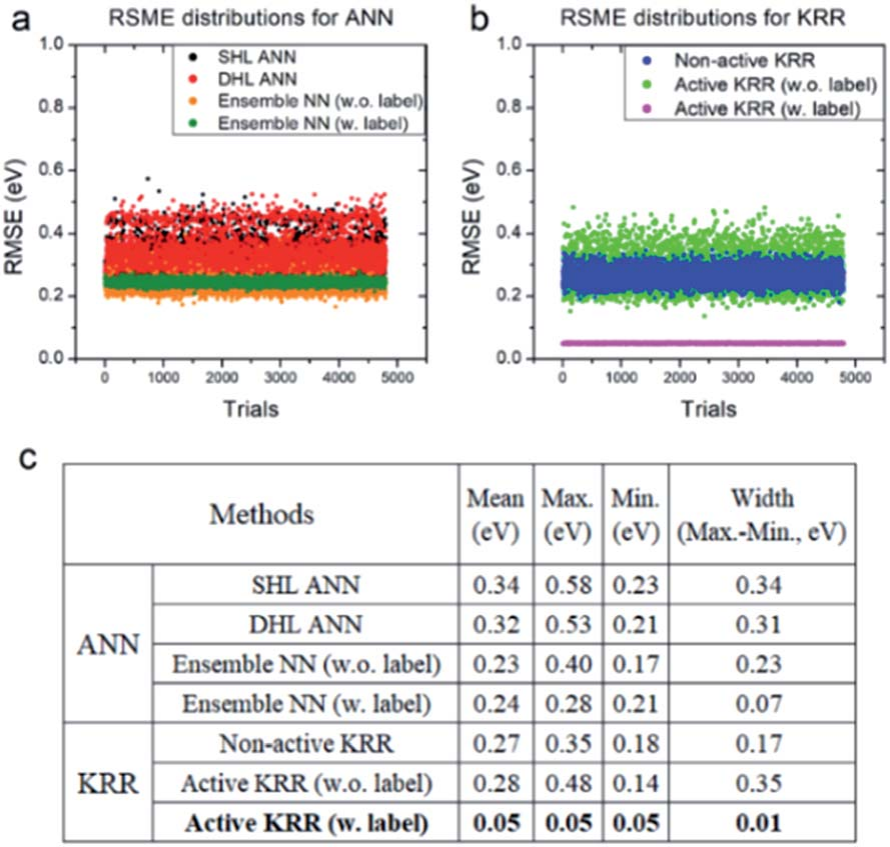
RMSE distributions for (a) ANN and (b) KRR, and (c) their detailed data for all of the trials. All of the statistics are for the machine learning model with the descriptors *W*LMTOd and *χ*_P_.

Next, we systematically analyzed the results with and without active learning techniques, which are summarized in Fig. 5 with the raw RMSE data of 4800 independent trials for ANN and KRR methods. For ANN, it is clear that the widths of the distributions are substantially reduced by applying an active learning algorithm from 0.31 eV for DHL ANN to 0.07 eV for ensemble NN (w. labels). Similar behavior is also seen in KRR, in which the width of distribution decreased from 0.17 eV for non-active KRR to 0.01 eV for active learning with labels. Interestingly, in the absence of the labels, the width increased with active learning (albeit still with improved final accuracy). Although, as shown in Fig. 5c, the effects of active learning are much more pronounced for KRR (0.18 → 0.05 eV) than for ANN (0.21 → 0.17 eV), it is possible that the use of different active learning algorithms for ANN other than the ensemble method used here may further enhance the resulting accuracy.

As a practical application of the actively learned chemisorption model with *W*LMTOd and *χ*_P_ as descriptors, we screened over 372 transition metal-based alloys (including 263 structures used in learning) with the structures shown in Fig. 1 to find active CRR catalysts to produce CO. Particularly, it has been suggested based on DFT calculations that the *CO binding energy is a key descriptor for the catalytic activity of CO_2_ reduction,^48^ and the current density measurements on various metal catalysts indeed showed a volcano-shaped relation of activity with respect to *CO binding energy.^50^ Currently, Au catalysts are reported to be the best single component catalyst for converting CO_2_ into CO, but alternative cost-effective catalysts are needed due to the high cost and scarcity of Au. To replace Au catalysts, one thus needs to develop catalysts with strong *E*_COOH_ to facilitate the activation of CO_2_, but not too strong *E*_CO_ so as to easily remove the product. Considering that the optimal *CO binding energy to achieve facile *COOH formation and *CO desorption is approximately –0.5 eV based on the scaling relation and the volcano plot,^48^ we selected candidates for which the *CO binding energies are in the range of –0.60 to –0.43 eV.^20^,^48^

**Fig. 6:**
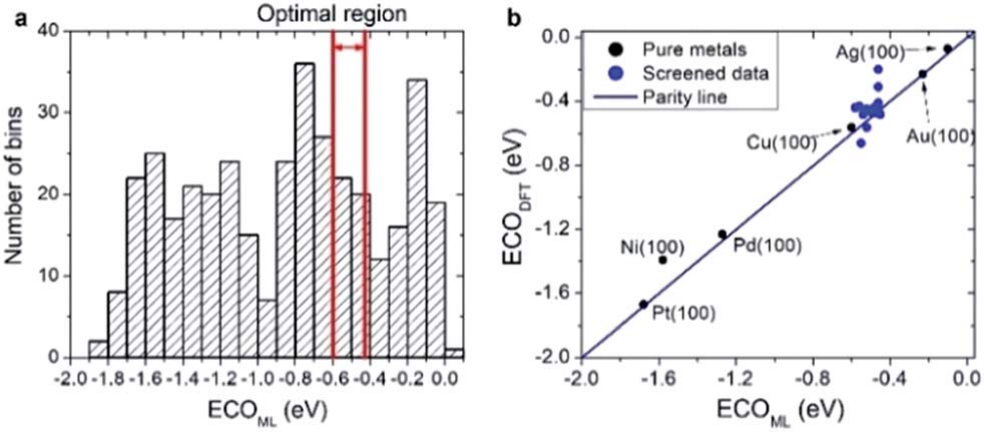
(a) A histogram of the predicted *CO binding energy (*E*_CO,ML_) using the active KRR machine using *W*LMTOd and *χ*_P_ as descriptors after screening 372 transition metal-based alloys. (b) The *CO binding energy comparison between the DFT calculation (*E*_CO,DFT_) and the prediction by machine learning (*E*_CO,ML_) for 15 candidates selected from (a).

As shown in Fig. 6a, our actively learned chemisorption model yielded 36 candidates within the optimal target window (–0.60 to –0.43 eV). Among them, we chose the alloys in which the outermost surface layer is covered by Cu or Au and that are nearest to the top of the volcano plot, yielding 15 candidates for further validation with good agreement between *E*_CO,DFT_ and *E*_CO,ML_ (see Fig. 6b). However, under experimental conditions complicated segregation/dissolution processes and mixing can occur in nanoalloys suggesting the importance of stability in catalyst design.^51^ In addition, the catalytic activity can even be enhanced by a change in surface metal composition, which could possibly be caused by favorable surface segregation under reaction conditions. For example, K. J. Andersson *et al.*^52^ experimentally investigated the surface segregation of a CuPt near-surface alloy (NSA) under CO adsorbed conditions. This indicates two main points: firstly, under an adsorption environment the electronic structure differs from vacuum conditions, and secondly, counterintuitively one can design a new type of alloy by inducing adsorption-driven surface segregation. In this context, we explored the stability of all 15 candidates under vacuum and a CO adsorbed state (details are shown in Fig. S1†) by changing the surface and subsurface layer. Herein, we use the symbol * for samples which are stable when the surface and subsurface layers are switched from the original structure shown in Fig. 1.

**Fig. 7:**
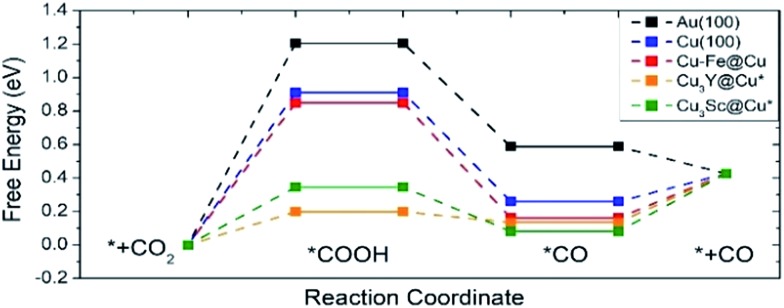
A free energy diagram of selected catalysts. Pure Au(100) and Cu(100) surfaces are also plotted as references. The symbol * indicates samples that are stable when the surface and subsurface layers are switched from the original structure shown in Fig. 1.

Fig. 7 shows the free energy diagram for selected catalysts (full results are found in Fig. S2†) including Au(100) and Cu(100) as references, and all of the selected catalysts show stabilized *COOH and *CO compared to Au(100) and Cu(100). Considering the limiting potential (*U*_L_ = –Δ*G*_MAX_/*e*) as a measure of CRR activity, the free energy diagram indicates that the *U*_L_ of Cu–Fe@Cu (–0.85 V) is less negative than that of Au(100) (–1.21 V) by 0.36 V. Furthermore, Cu_3_Y@Cu* and Cu_3_Sc@Cu* have a *U*_L_ of –0.20 V and –0.35 V respectively, which is less negative than Au(100) by 1.01 V and 0.86 V respectively. The catalytic activity of Cu_3_Y@Cu* is expected to outperform various Au-based catalysts, such as a Au–Cu bi-functional interfacial catalyst (*U*_L_ = –0.60 V)^53^ and Au NP corner site (*U*_L_ = –0.60 V).^54^ These results should also be compared to the experimental potentials of the best performing Au-based catalysts that reach a current density of CO production of more than 5 mA cm^–2^ in literature; –0.40 V for oxide-derived Au nanoparticles,^55^ –0.35 V for Au needles^56^ and –0.35 V for Au nanowires.^57^ All of these results imply that Cu_3_Y@Cu* could be highly active, comparable to the Au catalysts, and a cost-effective alternative to the Au catalysts for the CO_2_ reduction reaction.

## 5. Conclusions

We presented a machine learning model that can predict the binding energy of surface adsorbates on alloys using simple non-*ab initio* input features, namely, linear muffin-tin orbital theory (LMTO)-based d-band width and a geometric mean of electronegativity. By combining the aforementioned descriptors with the active learning algorithm, we obtained a high accuracy (RMSE = 0.05 eV for active KRR with labels) to predict *CO binding energy. The use of the LMTO d-band width as a learning descriptor yielded a higher prediction accuracy than the DFT-based d-band width due to the local characteristics of the *W*LMTOd. The effects of active learning were significant, lowering the RMSE for a neural network 0.21 → 0.17 eV, and for KRR 0.18 → 0.05 eV. The present d-band width model is also shown to work reasonably well for other facets such as (111) and (211) step sites to describe different coordination environments (with a combined error of 0.08 eV for all three facets). As an example of the practical application of the constructed KRR machine, we then screened the alloy catalysts for the CO_2_ electro-chemical reduction reaction by estimating their *CO binding energies, and identified that Cu_3_Sc@Cu* and Cu_3_Y@Cu* have an overpotential of ∼1 V lower than Au(100). We expect that the non-*ab initio* descriptors proposed here can be easily applicable to other types of catalyst designs, such as understanding the statistical behaviour of realistic experimental nanoparticles or nanowires with long temporal and large spatial sampling aspects where there are thousands of possible reaction sites with different local environments.^58^ Being able to rapidly estimate *CO binding energies using the easy-to-compute input features proposed here will undoubtedly be helpful to provide new insights for exciting experimental CO_2_ reduction results on complex surfaces.

## Conflicts of interest

There are no conflicts to declare.

## Supplementary Material

Supplementary information

## References

[cit1] Sabatier P. (1911). Eur. J. Inorg. Chem..

[cit2] Medford A. J., Vojvodic A., Hummelshøj J. S., Voss J., Abild-Pedersen F., Studt F., Bligaard T., Nilsson A., Nørskov J. K. (2015). J. Catal..

[cit3] Hammer B., Nørskov J. K. (2000). Adv. Catal..

[cit4] Nørskov J. K., Bligaard T., Rossmeisl J., Christensen C. H. (2009). Nat. Chem..

[cit5] Greeley J., Stephens I., Bondarenko A., Johansson T. P., Hansen H. A., Jaramillo T., Rossmeisl J., Chorkendorff I., Nørskov J. K. (2009). Nat. Chem..

[cit6] Back S., Jung Y. (2017). ChemCatChem.

[cit7] Back S., Kim H., Jung Y. (2015). ACS Catal..

[cit8] Stamenkovic V., Mun B. S., Mayrhofer K. J., Ross P. N., Markovic N. M., Rossmeisl J., Greeley J., Nørskov J. K. (2006). Angew. Chem..

[cit9] Gajdo M., Eichler A., Hafner J. (2004). J. Phys.: Condens. Matter.

[cit10] Xin H., Vojvodic A., Voss J., Nørskov J. K., Abild-Pedersen F. (2014). Phys. Rev. B: Condens. Matter Mater. Phys..

[cit11] Vojvodic A., Nørskov J. K., Abild-Pedersen F. (2014). Top. Catal..

[cit12] Jennings P. C., Lysgaard S., Hansen H. A., Vegge T. (2016). Phys. Chem. Chem. Phys..

[cit13] Calle-Vallejo F., Martinez J. I., Garcia-Lastra J. M., Sautet P., Loffreda D. (2014). Angew. Chem., Int. Ed..

[cit14] Calle-Vallejo F., Loffreda D., Koper M. T., Sautet P. (2015). Nat. Chem..

[cit15] Calle-Vallejo F., Tymoczko J., Colic V., Vu Q. H., Pohl M. D., Morgenstern K., Loffreda D., Sautet P., Schuhmann W., Bandarenka A. S. (2015). Science.

[cit16] Ma X., Xin H. (2017). Phys. Rev. Lett..

[cit17] Jain A., Ong S. P., Hautier G., Chen W., Richards W. D., Dacek S., Cholia S., Gunter D., Skinner D., Ceder G. (2013). APL Mater..

[cit18] Harrison W. A., Electronic structure and the properties of solids: the physics of the chemical bond, Courier Corporation, 2012.

[cit19] Xin H., Holewinski A., Linic S. (2012). ACS Catal..

[cit20] Ma X., Li Z., Achenie L. E., Xin H. (2015). J. Phys. Chem. Lett..

[cit21] Li Z., Ma X. F., Xin H. L. (2017). Catal. Today.

[cit22] Ulissi Z. W., Medford A. J., Bligaard T., Norskov J. K. (2017). Nat. Commun..

[cit23] Xin H., Linic S. (2010). J. Chem. Phys..

[cit24] Xin H., Schweitzer N., Nikolla E., Linic S. (2010). J. Chem. Phys..

[cit25] Xin H. L., Holewinski A., Schweitzer N., Nikolla E., Linic S. (2012). Top. Catal..

[cit26] Calle-Vallejo F., Pohl M. D., Reinisch D., Loffreda D., Sautet P., Bandarenka A. S. (2017). Chem. Sci..

[cit27] Van Ooyen A., Nienhuis B. (1992). Neural Network.

[cit28] Hansen K., Montavon G., Biegler F., Fazli S., Rupp M., Scheffler M., von Lilienfeld O. A., Tkatchenko A., Muller K. R. (2013). J. Chem. Theory Comput..

[cit29] Vu K., Snyder J. C., Li L., Rupp M., Chen B. F., Khelif T., Mueller K. R., Burke K. (2015). Int. J. Quantum Chem..

[cit30] Krogh A., Vedelsby J. (1995). Adv. Neural Inf. Process. Syst..

[cit31] Settles B., Synthesis Lectures on Artificial Intelligence and Machine Learning, 2012, vol. 6, pp. 1–114.

[cit32] O’Neill J., Delany S. J. and MacNamee B., in Advances in Computational Intelligence Systems, Springer, 2017, pp. 375–386.

[cit33] Sharma M., Bilgic M. (2017). Data Min. Knowl. Discov..

[cit34] Douak F., Melgani F., Benoudjit N. (2013). Appl. Energy.

[cit35] Yu K., Bi J. and Tresp V., Presented in part at the Proceedings of the 23rd international conference on Machine learning, Pittsburgh, Pennsylvania, USA, 2006.

[cit36] Demuth H. B. and Beale M. H., Neural Network Toolbox; for Use with MATLAB; Computation, Visualization, Programming; User’s Guide, Version 4, Math Works, 2000.

[cit37] Sharma G., Martin J. (2009). Int. J. Parallel Program..

[cit38] Levenberg K. (1944). Q. Appl. Math..

[cit39] Marquardt D. W. (1963). J. Soc. Ind. Appl. Math..

[cit40] Press W. H., Teukolsky S., Vetterling W. and Flannery B., Numerical Recipes in C, Cambridge University Press, 1988, vol. 1, p. 3.

[cit41] Kresse G., Furthmüller J. (1996). Phys. Rev. B: Condens. Matter Mater. Phys..

[cit42] Blöchl P. E. (1994). Phys. Rev. B: Condens. Matter Mater. Phys..

[cit43] Perdew J. P., Burke K., Ernzerhof M. (1996). Phys. Rev. Lett..

[cit44] Hammer B., Hansen L. B., Nørskov J. K. (1999). Phys. Rev. B: Condens. Matter Mater. Phys..

[cit45] Monkhorst H. J., Pack J. D. (1976). Phys. Rev. B: Condens. Matter Mater. Phys..

[cit46] Bahn S. R., Jacobsen K. W. (2002). Comput. Sci. Eng..

[cit47] Christensen R., Hansen H. A., Vegge T. (2015). Catal. Sci. Technol..

[cit48] Peterson A. A., Nørskov J. K. (2012). J. Phys. Chem. Lett..

[cit49] Kitchin J. R., Norskov J. K., Barteau M. A., Chen J. G. (2004). Phys. Rev. Lett..

[cit50] Kuhl K. P., Hatsukade T., Cave E. R., Abram D. N., Kibsgaard J., Jaramillo T. F. (2014). J. Am. Chem. Soc..

[cit51] Lysgaard S., Myrdal J. S., Hansen H. A., Vegge T. (2015). Phys. Chem. Chem. Phys..

[cit52] Andersson K. J., Calle-Vallejo F., Rossmeisl J., Chorkendorff I. (2009). J. Am. Chem. Soc..

[cit53] Back S., Kim J.-H., Kim Y.-T., Jung Y. (2016). ACS Appl. Mater. Interfaces.

[cit54] Back S., Yeom M. S., Jung Y. (2015). ACS Catal..

[cit55] Chen Y., Li C. W., Kanan M. W. (2012). J. Am. Chem. Soc..

[cit56] Liu M., Pang Y., Zhang B., De Luna P., Voznyy O., Xu J., Zheng X., Dinh C. T., Fan F., Cao C., de Arquer F. P., Safaei T. S., Mepham A., Klinkova A., Kumacheva E., Filleter T., Sinton D., Kelley S. O., Sargent E. H. (2016). Nature.

[cit57] Zhu W., Zhang Y. J., Zhang H., Lv H., Li Q., Michalsky R., Peterson A. A., Sun S. (2014). J. Am. Chem. Soc..

[cit58] Li M., Zhao Z., Cheng T., Fortunelli A., Chen C.-Y., Yu R., Zhang Q., Gu L., Merinov B., Lin Z., Zhu E., Yu T., Jia Q., Guo J., Zhang L., Goddard W. A., Huang Y., Duan X. (2016). Science.

