# Supplementary material for: Active learning with non-ab initio input features toward efficient CO2 reduction catalysts
Source: Chem Sci. 2018 Apr 17;9(23):5152–9. doi: 10.1039/c7sc03422a (PMC5998799; doi:10.1039/c7sc03422a)
Supplement: Supplementary file 1 [file SC-009-C7SC03422A-s001.pdf]

Electronic Supplementary Material (ESI) for Chemical Science.
This journal is © The Royal Society of Chemistry 2018

Electronic Supplementary Information (ESI) for

Actively learned machine with non-*ab initio* input features toward efficient CO_2 reduction catalyst

Juhwan Noh, Seoin Back, Jaehoon Kim and Yousung Jung*

Graduate School of EEWS, Korea Advanced Institute of Science and Technology (KAIST),

291 Daehakro, Daejeon 305-701, Korea

**E-mail: ysjn@kaist.ac.kr*

Brief overview of LMTO theory and d-band width calculation

In Muffin-Tin Orbital (MTO) theory, the potential in the crystal is approximated by spherically symmetric atomic potential within a sphere of radius r_{mt} (muffin-tin radius) and a constant potential region (muffin-tin potential) between the spherical atomic orbitals[1]. Based on this MTO concept, the interatomic coupling of d-states can be calculated as following expressions [1]:

$$V_{f(d,d)} = \sum_k \frac{\langle d' \mid \Delta \mid k\rangle\langle k \mid \Delta \mid d\rangle}{E_d - E_k} = \eta_{f(d,d)}\frac{\hbar^2 r_d^3}{m d_{ij}^5}, \tag{S1}$$

$$\Delta = \delta V - \langle d \mid \delta V \mid d\rangle, \tag{S2}$$

$$\delta V(r) = \begin{cases} 0, & r \le r_d \\ E_d - V_a(r), & r > r_d \end{cases}. \tag{S3}$$

In Eq. S1, r_d is a spatial extent of d-orbital taken from ref.[2], d_{ij} is an interatomic distance, and $f(d,d)$ is the classifier of interactions; for example, $\sigma(d,d)$ denotes the σ-interaction, and $\pi(d,d)$ denotes the π-interaction. Also, $\eta_{\sigma(d,d)}$, $\eta_{\pi(d,d)}$ and $\hbar^2/m$ are constant which can be taken from ref.[1]. Calculating Eq. S1 between different atom types can be done by changing r_d^3 to $r_{i,d}^{3/2} r_{j,d}^{3/2}$. Δ is a hybridization potential as shown in Eq. S2 indicating a constant shift of the potential difference function (δV), where δV is a potential difference between the constant d-state (E_d) and the potential made by nucleus ($V_a(r)$) as shown in Eq. S3. Finally, using Eq. S1 with additional mathematical treatments, the d-band width (W_d^{LMTO}) can be obtained as following [1]:

$$W_d^{LMTO} = -\frac{8}{3}V_{\sigma(d,d)}^{1st} + \frac{32}{9}V_{\pi(d,d)}^{1st} - 3V_{\sigma(d,d)}^{2nd} + 4V_{\pi(d,d)}^{2nd}. \tag{S4}$$

In Eq. S4, superscripts 1st and 2nd denote the first and second nearest neighbor atoms based on inter-atomic distances, and we calculated Eq. S4 layer by layer as shown in Eq. S5.

$$W_d^{LMTO} = W_{d,surf.}^{LMTO} + W_{d,sub.}^{LMTO} \tag{S5}$$

Furthermore, when considering $W_{d,surf.}^{LMTO}$, the lattice parameter of surface atom was used to calculate

interatomic distance, and when considering $W_{d,sub.}^{LMTO}$, the lattice parameter was obtained from Vegard's law [3] as shown in Eq. S6.

$$a_{M,X} = xa_M + (1 - x)a_X, \text{ where } x = n_M/(n_M + n_X) \quad \text{(S6)}$$

In Eq. S6, a_M is the lattice paramter of atom M, and a_X is the lattice parameter of atom X. In addition, n_M and n_X are the number of M and X existing in topmost two layers. Based on these, calculations of each term in Eq. S4 are following:

Table S1 Calculation of the interatomic coupling terms for the M_3X@M alloy structure.

M_3X@M	Surface	Sub-surface ($x = \frac{n_M}{n_M + n_X} = 0.75$)
$V_{\sigma(d,d)}^{1st}$	$4 * \eta_{\sigma(d,d)} \frac{\hbar^2 r_{d,M}^{3/2} r_{d,M}^{3/2}}{m a_M^5}$	$2 * \eta_{\pi(d,d)} \frac{\hbar^2 r_{d,M}^{\frac{3}{2}} r_{d,M}^{\frac{3}{2}}}{m a_{M,X}^5} + 2 * \eta_{\pi(d,d)} \frac{\hbar^2 r_{d,M}^{\frac{3}{2}} r_{d,X}^{\frac{3}{2}}}{m a_{M,X}^5}$
$V_{\sigma(d,d)}^{2nd}$	$4 * k_1 \eta_{\sigma(d,d)} \frac{\hbar^2 r_{d,M}^{3/2} r_{d,M}^{3/2}}{m(\sqrt{2} a_M)^5}$	$\frac{1}{2}\left[4 * \eta_{\sigma(d,d)} \frac{\hbar^2 r_{d,M}^{\frac{3}{2}} r_{d,M}^{\frac{3}{2}}}{m(\sqrt{3} a_{M,X})^5} + 4 * \eta_{\sigma(d,d)} \frac{\hbar^2 r_{d,M}^{\frac{3}{2}} r_{d,X}^{\frac{3}{2}}}{m(\sqrt{3} a_{M,X})^5}\right]$
$V_{\pi(d,d)}^{1st}$	$4 * \eta_{\pi(d,d)} \frac{\hbar^2 r_{d,M}^{3/2} r_{d,M}^{3/2}}{m a_M^5}$	$k_2\left[2 * \eta_{\pi(d,d)} \frac{\hbar^2 r_{d,M}^{\frac{3}{2}} r_{d,M}^{\frac{3}{2}}}{m a_{M,X}^5} + 2 * \eta_{\pi(d,d)} \frac{\hbar^2 r_{d,M}^{\frac{3}{2}} r_{d,X}^{\frac{3}{2}}}{m a_{M,X}^5}\right]$
$V_{\pi(d,d)}^{2nd}$	$4 * k_1 \eta_{\pi(d,d)} \frac{\hbar^2 r_{d,M}^{3/2} r_{d,M}^{3/2}}{m(\sqrt{2} a_M)^5}$	$\frac{k_2}{2}\left[4 * \eta_{\pi(d,d)} \frac{\hbar^2 r_{d,M}^{\frac{3}{2}} r_{d,M}^{\frac{3}{2}}}{m(\sqrt{3} a_{M,X})^5} + 4 * \eta_{\pi(d,d)} \frac{\hbar^2 r_{d,M}^{\frac{3}{2}} r_{d,X}^{\frac{3}{2}}}{m(\sqrt{3} a_{M,X})^5}\right]$

Table S2 Calculation of the interatomic coupling terms for the M-X@M and X@M alloy structures.

M-X@M and X@M	Surface	Sub-surface ($x = \frac{n_M}{n_M + n_X} = 0.5$)
$V_{\sigma(d,d)}^{1st}$	$4 * \eta_{\sigma(d,d)} \frac{\hbar^2 r_{d,M}^{3/2} r_{d,M}^{3/2}}{m a_M^5}$	$4 * \eta_{\sigma(d,d)} \frac{\hbar^2 r_{d,M}^{\frac{3}{2}} r_{d,X}^{\frac{3}{2}}}{m a_{M,X}^5}$

$V_{\sigma(d,d)}^{2nd}$	$4*k_1\eta_{\sigma(d,d)}\frac{\hbar^2 r_{d,M}^{3/2} r_{d,M}^{3/2}}{m(\sqrt{2}a_M)^5}$	$\frac{1}{2}\left[8*\eta_{\sigma(d,d)}\frac{\hbar^2 r_{d,M}^{\frac{3}{2}} r_{d,X}^{\frac{3}{2}}}{m(\sqrt{3}a_{M,X})^5}\right]$
$V_{\pi(d,d)}^{1st}$	$4*\eta_{\pi(d,d)}\frac{\hbar^2 r_{d,M}^{3/2} r_{d,M}^{3/2}}{m a_M^5}$	$k_2\left[4*\eta_{\pi(d,d)}\frac{\hbar^2 r_{d,M}^{\frac{3}{2}} r_{d,X}^{\frac{3}{2}}}{m a_{M,X}^5}\right]$
$V_{\pi(d,d)}^{2nd}$	$4*k_1\eta_{\pi(d,d)}\frac{\hbar^2 r_{d,M}^{3/2} r_{d,M}^{3/2}}{m(\sqrt{2}a_M)^5}$	$\frac{k_2}{2}\left[8*\eta_{\pi(d,d)}\frac{\hbar^2 r_{d,M}^{\frac{3}{2}} r_{d,X}^{\frac{3}{2}}}{m(\sqrt{3}a_{M,X})^5}\right]$

In the tables, $r_{d,M}$ and $r_{d,X}$ are the spatial extents of d-state for atom M and X, respectively, and for both k_1 and k_2 we explicitly used $k_1 = 16$ and $k_2 =- 1.85$. In addition, all multiplied integers denote the number of coordinated atoms.

Stability analysis and free energy diagram for screened 15-candidates

As mentioned in main text, to explore stability of the screened 15-candidates we calculated segregation energy ($E_{seg.}$, in eV) under vacuum ($E_{seg.}^{vac.}$) and CO adsorbed state ($E_{seg.}^{*CO}$), and $E_{seg.}$ is defined as energy difference after switching surface and subsurface layers from original structure shown in Fig. 1 in the main text.

a

Samples with -0.60 eV < ECO ≤ -0.43 eV									
$Cu_3X@Cu$	Mo	Nb	Re	Sc	Ta	Ti	W	Y	Zr
Cu-X@Cu	Fe								
X@Au	Pt	Rh							
Au-X@Au	Pt	Rh	Ru						

b

Stability classification results									
$Cu_3X@Cu$	Mo (2.36/0.92)	Nb (0.96/-0.08)	Re (3.96/2.06)	Sc (-1.57/-1.85)	Ta (1.68/0.68)	Ti (0.48/-0.20)	W (3.49/1.88)	Y (-3.64/-3.87)	Zr (-1.24/-1.78)
Cu-X@Cu	Fe (2.55/1.47)								
X@Au	Pt (4.58/3.60)	Rh (8.51/6.91)						[Legend] X ($E^{vac.}_{seg.}/E^{*CO}_{seg.}$)	
Au-X@Au	Pt (3.86/2.52)	Rh (5.15/3.61)	Ru (6.75/5.02)						

Fig. S 1 Stability analysis upon reaction (adsorption adsorption) condition: (a) List of the selected 15-candidates as a result of ML-based screening with proper *CO binding energy. (b) Calculation results for segregation energy (E_{seg}) under vacuum ($E^{vac.}_{seg.}$) and CO adsorbated state ($E^{*CO}_{seg.}$). Yellow color means that the sample is stable with original configurations shown in Fig 1 under both vacuum and CO adsorbed state, blue color means that the sample is stable when the surface and subsurfaces are switched under both vacuum and CO adsorbed state, and green color means that the sample changes its configurations upon the gas adsorption.

Using the stability results of the surfaces from Fig. S1, we calculated the free energy for CO production process using the most stable surface configurations upon reaction condition, as summarized in Fig. S2.

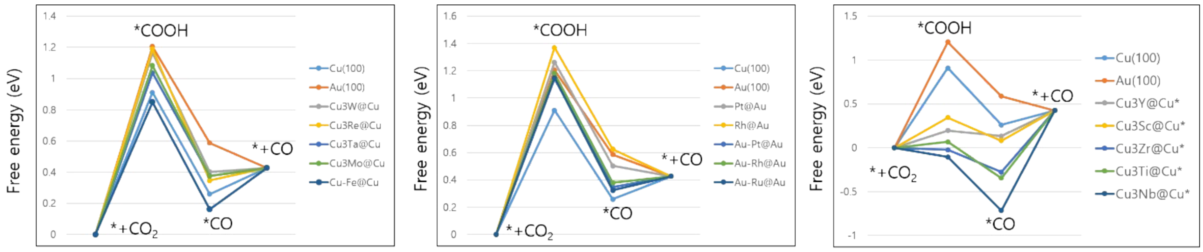

Fig. S 2 Free energy diagrams for alloy catalysts using the most stable surface configurations upon reaction condition: the symbol * in Cu3Y@Cu*, for example, is used for samples which are stable with the surface and subsurfaces switched under CO adsorbated state.

Table S3 Descriptors used for machine learning models

Alloy name	This work			Taken from the ref.[4]		
	χ_M	χ_P	W_d^{LMTO} (eV)	W_d^{cal} (eV)	d_c (eV)	ECO_{cal} (eV)
Ag3Ag@Ag	4.44	1.93	31.37	1.10	-3.92	-0.09
Ag3Au@Ag	4.74	2.07	31.19	1.13	-3.86	-0.11
Ag3Co@Ag	4.40	1.92	30.56	1.19	-3.90	-0.11
Ag3Cr@Ag	4.25	1.86	30.58	1.13	-4.03	-0.12
Ag3Cu@Ag	4.45	1.92	31.43	1.16	-3.99	-0.08
Ag3Ir@Ag	4.65	1.99	30.94	1.22	-3.66	-0.14
Ag3Mn@Ag	4.25	1.83	32.42	1.15	-4.08	-0.12
Ag3Nb@Ag	4.28	1.84	30.14	1.14	-3.89	-0.07
Ag3Ni@Ag	4.43	1.92	31.33	1.20	-3.85	-0.09
Ag3Os@Ag	4.55	1.99	29.94	1.24	-3.74	-0.10
Ag3Pd@Ag	4.44	1.99	31.19	1.13	-3.69	-0.08
Ag3Pt@Ag	4.70	2.01	31.05	1.18	-3.68	-0.12
Ag3Ru@Ag	4.38	1.99	30.09	1.18	-3.65	-0.11
Ag3Sc@Ag	4.15	1.77	30.24	1.06	-4.02	-0.05
Ag3Ta@Ag	4.36	1.81	30.02	1.21	-3.98	-0.02
Ag3Ti@Ag	4.17	1.82	30.26	1.09	-4.01	-0.10
Ag3V@Ag	4.22	1.85	30.53	1.13	-3.98	-0.12
Ag3W@Ag	4.43	2.03	30.03	1.24	-3.89	-0.03
Au3Ag@Au	5.40	2.37	46.62	1.37	-3.17	-0.21
Au3Au@Au	5.77	2.54	46.40	1.41	-3.18	-0.26
Au3Cu@Au	5.42	2.36	46.67	1.48	-3.34	-0.17
Au3Pd@Au	5.41	2.45	46.39	1.43	-2.99	-0.30
Au3Pt@Au	5.72	2.47	46.22	1.47	-3.02	-0.36
Cu3Ag@Cu	4.48	1.91	24.88	0.96	-2.09	-0.70
Cu3Au@Cu	4.78	2.04	24.70	1.02	-2.11	-0.72
Cu3Co@Cu	4.43	1.89	24.14	1.07	-2.19	-0.63
Cu3Cr@Cu	4.28	1.84	24.12	1.01	-2.36	-0.66
Cu3Cu@Cu	4.49	1.90	24.98	1.06	-2.26	-0.63
Cu3Ir@Cu	4.69	1.97	24.45	1.10	-2.09	-0.72
Cu3La@Cu	4.17	1.66	23.41	0.86	-2.16	-0.60
Cu3Mn@Cu	4.28	1.81	25.83	1.01	-2.38	-0.63
Cu3Mo@Cu	4.34	1.96	23.68	1.03	-2.27	-0.48
Cu3Nb@Cu	4.32	1.82	23.64	0.99	-2.36	-0.51
Cu3Ni@Cu	4.47	1.90	24.88	1.07	-2.15	-0.66
Cu3Os@Cu	4.59	1.97	23.44	1.13	-2.23	-0.65
Cu3Pd@Cu	4.48	1.97	24.71	1.01	-1.94	-0.70

Cu3Pt@Cu	4.74	1.99	24.56	1.07	-1.97	-0.74
Cu3Re@Cu	4.37	1.90	23.28	1.13	-2.31	-0.49
Cu3Rh@Cu	4.44	1.99	24.58	1.04	-1.98	-0.72
Cu3Ru@Cu	4.42	1.97	23.61	1.04	-2.04	-0.67
Cu3Sc@Cu	4.18	1.75	23.73	0.91	-2.38	-0.51
Cu3Ta@Cu	4.39	1.79	23.51	1.03	-2.47	-0.49
Cu3Ti@Cu	4.21	1.80	23.77	0.97	-2.45	-0.52
Cu3V@Cu	4.26	1.83	24.06	1.00	-2.34	-0.65
Cu3W@Cu	4.47	2.01	23.52	1.08	-2.38	-0.46
Cu3Y@Cu	4.15	1.70	23.31	0.90	-2.31	-0.49
Cu3Zr@Cu	4.23	1.74	23.26	0.93	-2.39	-0.56
Ni3Ag@Ni	4.41	1.91	33.75	1.22	-1.10	-1.62
Ni3Au@Ni	4.71	2.05	33.53	1.27	-1.12	-1.68
Ni3Co@Ni	4.37	1.90	32.79	1.36	-1.42	-1.47
Ni3Cr@Ni	4.22	1.84	32.79	1.33	-1.31	-1.53
Ni3Cu@Ni	4.42	1.91	33.85	1.29	-1.19	-1.52
Ni3Ir@Ni	4.62	1.98	33.22	1.37	-1.30	-1.58
Ni3La@Ni	4.10	1.66	31.98	1.07	-0.75	-1.47
Ni3Mo@Ni	4.27	1.97	32.27	1.37	-1.52	-1.36
Ni3Nb@Ni	4.25	1.83	32.23	1.34	-1.49	-1.35
Ni3Ni@Ni	4.40	1.91	33.73	1.33	-1.32	-1.52
Ni3Os@Ni	4.52	1.98	31.96	1.43	-1.45	-1.44
Ni3Pd@Ni	4.41	1.98	33.53	1.27	-1.20	-1.61
Ni3Pt@Ni	4.67	2.00	33.36	1.33	-1.25	-1.65
Ni3Re@Ni	4.30	1.91	31.77	1.46	-1.59	-1.34
Ni3Rh@Ni	4.37	2.00	33.37	1.32	-1.30	-1.56
Ni3Ru@Ni	4.35	1.98	32.16	1.32	-1.33	-1.52
Ni3Sc@Ni	4.12	1.75	32.34	1.18	-0.94	-1.47
Ni3Ta@Ni	4.33	1.80	32.07	1.40	-1.54	-1.33
Ni3Ti@Ni	4.14	1.81	32.37	1.30	-1.20	-1.52
Ni3V@Ni	4.20	1.84	32.72	1.37	-1.52	-1.40
Ni3W@Ni	4.40	2.01	32.08	1.43	-1.60	-1.34
Ni3Y@Ni	4.09	1.71	31.85	1.10	-0.79	-1.49
Ni3Zr@Ni	4.16	1.74	31.77	1.23	-1.04	-1.54
Pd3Ag@Pd	4.45	2.13	47.73	1.29	-1.53	-1.24
Pd3Au@Pd	4.75	2.28	47.50	1.35	-1.54	-1.34
Pd3Co@Pd	4.40	2.12	46.55	1.48	-1.95	-1.07
Pd3Cr@Pd	4.26	2.05	46.60	1.43	-1.87	-0.99
Pd3Cu@Pd	4.46	2.12	47.79	1.40	-1.72	-1.15
Pd3Ir@Pd	4.66	2.20	47.15	1.50	-1.93	-1.20

Pd3La@Pd	4.14	1.85	45.84	1.25	-1.48	-0.80
Pd3Mn@Pd	4.26	2.02	49.19	1.41	-1.75	-1.00
Pd3Mo@Pd	4.31	2.19	46.10	1.56	-2.36	-0.94
Pd3Nb@Pd	4.29	2.03	46.07	1.58	-2.59	-0.80
Pd3Ni@Pd	4.44	2.12	47.65	1.43	-1.82	-1.17
Pd3Os@Pd	4.56	2.20	45.74	1.59	-2.20	-1.08
Pd3Pd@Pd	4.45	2.20	47.48	1.36	-1.67	-1.28
Pd3Pt@Pd	4.71	2.22	47.30	1.42	-1.72	-1.31
Pd3Re@Pd	4.34	2.12	45.55	1.65	-2.45	-0.93
Pd3Rh@Pd	4.41	2.22	47.31	1.41	-1.82	-1.24
Pd3Sc@Pd	4.15	1.95	46.19	1.39	-1.72	-0.82
Pd3Ta@Pd	4.36	2.00	45.91	1.67	-2.76	-0.76
Pd3Ti@Pd	4.18	2.01	46.18	1.51	-2.27	-0.94
Pd3V@Pd	4.23	2.04	46.55	1.51	-2.19	-0.95
Pd3W@Pd	4.44	2.24	45.91	1.68	-2.67	-0.83
Pd3Y@Pd	4.12	1.90	45.70	1.31	-1.51	-0.82
Pd3Zr@Pd	4.20	1.94	45.59	1.51	-2.17	-0.89
Pt3Ag@Pt	5.26	2.19	62.37	1.74	-1.79	-1.58
Pt3Au@Pt	5.62	2.34	62.11	1.77	-1.80	-1.72
Pt3Co@Pt	5.21	2.17	60.90	1.95	-2.25	-1.44
Pt3Cr@Pt	5.04	2.11	61.00	1.92	-2.23	-1.39
Pt3Cu@Pt	5.28	2.18	62.41	1.88	-2.03	-1.44
Pt3Ir@Pt	5.51	2.26	61.70	1.95	-2.18	-1.62
Pt3La@Pt	4.90	1.90	60.21	1.54	-1.56	-1.25
Pt3Mo@Pt	5.10	2.25	60.46	2.00	-2.55	-1.32
Pt3Nb@Pt	5.07	2.09	60.44	2.01	-2.59	-1.20
Pt3Ni@Pt	5.25	2.18	62.24	1.91	-2.16	-1.51
Pt3Os@Pt	5.39	2.26	60.02	2.03	-2.40	-1.51
Pt3Pd@Pt	5.27	2.26	62.08	1.81	-1.97	-1.65
Pt3Pt@Pt	5.57	2.28	61.88	1.87	-2.03	-1.70
Pt3Re@Pt	5.13	2.18	59.80	2.10	-2.58	-1.38
Pt3Rh@Pt	5.22	2.28	61.88	1.87	-2.12	-1.61
Pt3Ru@Pt	5.19	2.26	60.23	1.91	-2.22	-1.57
Pt3Sc@Pt	4.92	2.00	60.58	1.83	-2.00	-1.23
Pt3Ta@Pt	5.16	2.05	60.26	2.11	-2.73	-1.15
Pt3Ti@Pt	4.94	2.07	60.54	1.96	-2.45	-1.31
Pt3V@Pt	5.01	2.10	60.95	1.98	-2.47	-1.37
Pt3W@Pt	5.26	2.30	60.25	2.13	-2.72	-1.18
Pt3Y@Pt	4.88	1.95	60.05	1.68	-1.71	-1.24
Pt3Zr@Pt	4.97	1.99	59.89	1.89	-2.19	-1.33

Ag-Ag@Ag	4.44	1.93	31.37	1.10	-3.92	-0.09
Ag-Au@Ag	5.06	2.21	31.01	1.17	-3.86	-0.12
Ag-Cu@Ag	4.46	1.91	31.56	1.18	-4.01	-0.06
Ag-La@Ag	3.85	1.46	28.21	1.06	-4.15	-0.12
Ag-Nb@Ag	4.12	1.76	28.31	1.26	-4.33	-0.24
Ag-Ni@Ag	4.42	1.92	31.33	1.20	-3.73	-0.14
Ag-Pd@Ag	4.44	2.06	30.99	1.13	-3.50	-0.15
Ag-Pt@Ag	4.97	2.10	30.70	1.23	-3.55	-0.23
Ag-Sc@Ag	3.87	1.62	28.55	1.17	-4.49	-0.06
Ag-Ta@Ag	4.27	1.70	28.01	1.35	-4.41	-0.27
Ag-Ti@Ag	3.92	1.72	28.36	1.16	-4.41	-0.15
Ag-Y@Ag	3.81	1.53	27.89	1.14	-4.36	-0.11
Ag-Zr@Ag	3.96	1.60	27.31	1.21	-4.42	-0.13
Au-Ag@Au	5.06	2.21	46.84	1.32	-3.19	-0.18
Au-Au@Au	5.77	2.54	46.40	1.41	-3.18	-0.26
Au-Cu@Au	5.09	2.20	47.07	1.45	-3.40	-0.11
Au-La@Au	4.38	1.67	43.00	1.36	-3.71	-0.11
Au-Pd@Au	5.07	2.36	46.38	1.39	-2.89	-0.39
Au-Pt@Au	5.67	2.41	46.03	1.49	-3.01	-0.51
Au-Ru@Au	4.93	2.36	42.34	1.49	-3.35	-0.47
Au-Y@Au	4.34	1.76	42.61	1.47	-4.01	-0.10
Cu-Ag@Cu	4.46	1.91	24.84	0.98	-2.13	-0.64
Cu-Au@Cu	5.09	2.20	24.52	1.04	-2.08	-0.67
Cu-Co@Cu	4.38	1.89	22.67	1.08	-2.34	-0.76
Cu-Cr@Cu	4.09	1.78	22.82	1.10	-2.59	-1.17
Cu-Cu@Cu	4.49	1.90	24.98	1.06	-2.26	-0.63
Cu-Ir@Cu	4.90	2.04	24.00	1.16	-2.31	-0.78
Cu-La@Cu	3.87	1.45	21.99	0.93	-2.41	-0.69
Cu-Mo@Cu	4.20	2.03	21.94	1.11	-2.52	-1.18
Cu-Nb@Cu	4.15	1.74	21.99	1.08	-2.71	-1.05
Cu-Ni@Cu	4.44	1.90	24.77	1.07	-2.14	-0.72
Cu-Os@Cu	4.69	2.04	20.85	1.19	-2.52	-0.90
Cu-Pd@Cu	4.47	2.04	24.49	1.03	-1.99	-0.75
Cu-Pt@Cu	5.00	2.08	24.24	1.12	-2.09	-0.79
Cu-Re@Cu	4.25	1.90	20.43	1.21	-2.64	-1.12
Cu-Rh@Cu	4.39	2.08	24.23	1.07	-2.14	-0.81
Cu-Ru@Cu	4.35	2.04	21.26	1.08	-2.31	-0.87
Cu-Sc@Cu	3.90	1.61	22.22	0.99	-2.72	-0.63
Cu-Ta@Cu	4.30	1.69	21.72	1.14	-2.83	-1.01
Cu-Ti@Cu	3.94	1.71	21.97	1.02	-2.83	-0.69

Cu-V@Cu	4.04	1.76	22.75	1.07	-2.74	-0.81
Cu-W@Cu	4.45	2.12	21.58	1.19	-2.63	-1.18
Cu-Y@Cu	3.83	1.52	21.69	0.96	-2.56	-0.73
Cu-Zr@Cu	3.98	1.59	21.06	1.02	-2.74	-0.84
Ni-Ag@Ni	4.42	1.92	33.81	1.18	-1.08	-1.61
Ni-Au@Ni	5.04	2.20	33.45	1.24	-1.08	-1.71
Ni-Co@Ni	4.33	1.89	31.24	1.37	-1.44	-1.45
Ni-Cr@Ni	4.05	1.78	31.44	1.38	-1.53	-1.39
Ni-Cu@Ni	4.44	1.90	33.97	1.26	-1.11	-1.54
Ni-Ir@Ni	4.85	2.05	32.84	1.45	-1.36	-1.80
Ni-Mo@Ni	4.15	2.03	30.41	1.40	-1.35	-1.65
Ni-Nb@Ni	4.11	1.75	30.49	1.37	-1.27	-1.47
Ni-Ni@Ni	4.40	1.91	33.73	1.33	-1.32	-1.52
Ni-Os@Ni	4.64	2.05	29.12	1.53	-1.50	-1.87
Ni-Pd@Ni	4.42	2.05	33.41	1.27	-1.22	-1.68
Ni-Pt@Ni	4.95	2.09	33.12	1.36	-1.28	-1.74
Ni-Re@Ni	4.21	1.90	28.63	1.54	-1.50	-1.75
Ni-Rh@Ni	4.35	2.09	33.10	1.34	-1.30	-1.73
Ni-Ru@Ni	4.30	2.05	29.60	1.36	-1.35	-1.87
Ni-Sc@Ni	3.86	1.61	30.75	1.28	-1.22	-1.27
Ni-Ta@Ni	4.25	1.69	30.17	1.43	-1.35	-1.44
Ni-Ti@Ni	3.90	1.72	30.44	1.33	-1.36	-1.30
Ni-V@Ni	4.00	1.76	31.36	1.36	-1.43	-1.36
Ni-W@Ni	4.41	2.12	30.00	1.50	-1.43	-1.62
Ni-Y@Ni	3.79	1.53	30.15	1.20	-1.10	-1.23
Ni-Zr@Ni	3.94	1.59	29.40	1.27	-1.19	-1.33
Pd-Ag@Pd	4.44	2.06	47.95	1.28	-1.40	-1.16
Pd-Au@Pd	5.07	2.36	47.51	1.39	-1.51	-1.31
Pd-Co@Pd	4.36	2.03	45.16	1.47	-2.06	-0.90
Pd-Cu@Pd	4.47	2.04	48.17	1.38	-1.64	-1.12
Pd-Ir@Pd	4.87	2.20	46.81	1.63	-2.23	-1.23
Pd-La@Pd	3.85	1.56	44.04	1.32	-1.96	-0.50
Pd-Mn@Pd	4.07	1.85	49.67	1.49	-2.03	-0.68
Pd-Mo@Pd	4.18	2.18	44.06	1.61	-2.46	-0.79
Pd-Nb@Pd	4.13	1.88	44.11	1.62	-2.47	-0.72
Pd-Ni@Pd	4.42	2.05	47.89	1.43	-1.90	-1.05
Pd-Os@Pd	4.67	2.20	42.68	1.71	-2.50	-1.05
Pd-Pd@Pd	4.45	2.20	47.48	1.36	-1.67	-1.28
Pd-Pt@Pd	4.98	2.24	47.13	1.49	-1.82	-1.33
Pd-Rh@Pd	4.37	2.24	47.12	1.47	-1.99	-1.18

Pd-Ru@Pd	4.33	2.20	43.24	1.51	-2.16	-1.09
Pd-Sc@Pd	3.88	1.73	44.42	1.50	-2.39	-0.48
Pd-Ta@Pd	4.28	1.82	43.74	1.71	-2.59	-0.74
Pd-Ti@Pd	3.92	1.84	44.14	1.51	-2.56	-0.53
Pd-V@Pd	4.02	1.89	45.18	1.54	-2.53	-0.61
Pd-W@Pd	4.43	2.28	43.58	1.75	-2.67	-0.80
Pd-Y@Pd	3.81	1.64	43.64	1.41	-2.17	-0.55
Pd-Zr@Pd	3.96	1.71	42.86	1.55	-2.32	-0.62
Pt-Ag@Pt	4.97	2.10	62.82	1.62	-1.56	-1.52
Pt-Au@Pt	5.67	2.41	62.32	1.74	-1.63	-1.67
Pt-Co@Pt	4.88	2.07	59.65	1.81	-2.12	-1.18
Pt-Cu@Pt	5.00	2.08	63.07	1.74	-1.76	-1.44
Pt-Ir@Pt	5.45	2.24	61.51	2.01	-2.41	-1.52
Pt-La@Pt	4.31	1.58	58.36	1.69	-2.05	-0.72
Pt-Mo@Pt	4.67	2.22	58.39	2.01	-2.59	-1.09
Pt-Nb@Pt	4.62	1.91	58.45	2.02	-2.60	-1.06
Pt-Ni@Pt	4.95	2.09	62.75	1.80	-2.05	-1.36
Pt-Os@Pt	5.22	2.24	56.83	2.10	-2.65	-1.40
Pt-Pd@Pt	4.98	2.24	62.28	1.71	-1.86	-1.64
Pt-Pt@Pt	5.57	2.28	61.88	1.87	-2.03	-1.70
Pt-Re@Pt	4.73	2.08	56.19	2.14	-2.73	-1.20
Pt-Rh@Pt	4.89	2.28	61.87	1.84	-2.19	-1.49
Pt-Ru@Pt	4.84	2.24	57.47	1.88	-2.32	-1.43
Pt-Sc@Pt	4.34	1.76	58.80	1.90	-2.46	-0.68
Pt-Ta@Pt	4.78	1.85	58.02	2.15	-2.74	-1.04
Pt-Ti@Pt	4.39	1.87	58.49	1.90	-2.70	-0.77
Pt-V@Pt	4.50	1.93	59.67	1.93	-2.65	-0.88
Pt-W@Pt	4.96	2.32	57.85	2.16	-2.76	-1.08
Pt-Y@Pt	4.27	1.67	57.90	1.80	-2.33	-0.79
Pt-Zr@Pt	4.43	1.74	57.02	1.95	-2.51	-0.89
Ag@Ag	4.44	1.93	31.37	1.10	-3.92	-0.09
Ag@Au	5.06	2.21	46.84	1.34	-3.21	-0.15
Ag@Cu	4.46	1.91	24.84	0.88	-1.90	-0.74
Ag@Ni	4.42	1.92	33.81	1.05	-0.77	-1.73
Ag@Pd	4.44	2.06	47.95	1.23	-1.31	-1.12
Ag@Pt	4.97	2.10	62.82	1.52	-1.38	-1.59
Au@Ag	5.06	2.21	31.01	1.16	-3.87	-0.15
Au@Au	5.77	2.54	46.40	1.41	-3.18	-0.26
Au@Cu	5.09	2.20	24.52	1.02	-1.98	-0.86
Au@Ni	5.04	2.20	33.45	1.19	-0.87	-1.80

Au@Pd	5.07	2.36	47.51	1.35	-1.35	-1.29
Au@Pt	5.67	2.41	62.32	1.64	-1.46	-1.84
Cu@Au	5.09	2.20	47.07	1.90	-3.88	-0.66
Cu@Cu	4.49	1.90	24.98	1.05	-2.25	-0.63
Cu@Ni	4.44	1.90	33.97	1.22	-1.01	-1.60
Cu@Pd	4.47	2.04	48.17	1.62	-2.05	-0.84
Cu@Pt	5.00	2.08	63.07	2.05	-2.33	-1.11
Ir@Ag	4.87	2.06	30.45	1.36	-3.81	-0.25
Ir@Au	5.55	2.36	45.72	1.72	-3.41	-0.37
Ir@Cu	4.90	2.04	24.00	1.13	-2.10	-0.91
Ir@Ni	4.85	2.05	32.84	1.36	-1.21	-1.59
Ir@Pd	4.87	2.20	46.81	1.60	-2.15	-1.18
Ir@Pt	5.45	2.24	61.51	2.04	-2.37	-1.49
Ni@Cu	4.44	1.90	24.77	1.11	-2.21	-0.69
Ni@Ni	4.40	1.91	33.73	1.33	-1.32	-1.52
Pd@Ag	4.44	2.06	30.99	1.22	-3.64	-0.14
Pd@Au	5.07	2.36	46.38	1.52	-3.07	-0.27
Pd@Cu	4.47	2.04	24.49	0.97	-1.66	-0.86
Pd@Ni	4.42	2.05	33.41	1.22	-1.13	-1.64
Pd@Pd	4.45	2.20	47.48	1.36	-1.67	-1.28
Pd@Pt	4.98	2.24	62.28	1.74	-1.90	-1.66
Pt@Ag	4.97	2.10	30.70	1.28	-3.69	-0.24
Pt@Au	5.67	2.41	46.03	1.59	-3.17	-0.40
Pt@Cu	5.00	2.08	24.24	1.09	-1.85	-0.91
Pt@Ni	4.95	2.09	33.12	1.34	-1.24	-1.63
Pt@Pd	4.98	2.24	47.13	1.48	-1.82	-1.31
Pt@Pt	5.57	2.28	61.88	1.87	-2.03	-1.70

References

1. W. A. Harrison, *Electronic structure and the properties of solids: the physics of the chemical bond*, Courier Corporation, 2012.
2. J. M. Wills and W. A. Harrison, *Physical Review B*, 1983, **28**, 4363-4373.
3. H. Xin, A. Holewinski and S. Linic, *ACS Catalysis*, 2012, **2**, 12-16.
4. X. Ma, Z. Li, L. E. Achenie and H. Xin, *J Phys Chem Lett*, 2015, **6**, 3528-3533.
